# How to predict the culprit segment in percutaneous transforaminal endoscopic surgery under local anesthesia for surgical treatment of lumbar degenerative diseases? Radiologic images or clinical symptoms

**DOI:** 10.3389/fsurg.2022.1060318

**Published:** 2023-01-06

**Authors:** Tianyao Zhou, Tianle Ma, Yutong Gu, Liang Zhang, Wu Che, Yichao Wang

**Affiliations:** ^1^ Department of Orthopedic Surgery, Zhongshan Hospital Fudan University, Shanghai, China; ^2^ Shanghai Southwest Spine Surgery Center, Shanghai, China

**Keywords:** lumbar degenerative disease, culprit segment, radiologic images, clinical symptoms, transforaminal endoscopic discectomy, minimally invasive surgery

## Abstract

**Objective:**

Percutaneous transforaminal endoscopic surgery (PTES) is a novel, minimally invasive technique used to treat lumbar degenerative diseases (LDDs). PTES under local anesthesia was performed to treat the culprit segment of LDDs predicted by radiologic images or clinical symptoms, and the efficacy, security, and feasibility were evaluated.

**Methods:**

Eighty-seven cases of LDDs with nerve root symptoms, which were not consistent with lumbar degenerative levels and degrees on MRI and CT, were treated with PTES under local anesthesia in a day surgery ward from January 2015 to December 2019. Forty-two patients, whose culprit segments were predicted by radiologic images, were included in group A. The other 45 patients, whose culprit segments were predicted by clinical symptoms, were included in group B. Leg pain VAS and ODI scores before and after PTES were recorded. The outcome was defined according to the MacNab grade at the 2-year follow-up. Postoperative complications were recorded.

**Results:**

In group A, 2 patients underwent PTES for one segment, 37 patients underwent PTES for two segments, and 3 patients underwent PTES for three segments. One of the one-segment PTES patients had no relief from symptoms and underwent another PTES for other culprit segments 3 months after surgery. In group B, 44 of 45 patients were treated using PTES for one segment and 1 patient was treated for two segments. Group B showed significantly less operative duration, less blood loss, and less fluoroscopy frequency than group A (*p* < 0.001). The leg pain VAS score and the ODI score significantly decreased after the operation in both groups (*p* < 0.001), and the excellent and good rates were 97.6% (41/42) in group A and 100% (45/45) in group B at the 2-year follow-up. The leg pain VAS score of group B was significantly lower than that of group A immediately and 1 week, 1 month, 2 months, and 3 months after surgery (*p* < 0.001). There was no statistical difference in ODI scores and the excellent and good rates between the two groups. No complications, such as wound infection or permanent nerve injury, were observed.

**Conclusion:**

It is much more accurate to predict the culprit segment according to clinical symptoms than radiologic images in PTES under local anesthesia for surgical treatment of LDDs.

## Introduction

With the extension of life, lumbar degenerative diseases (LDDs) are becoming more prevalent (1, 2). The neurologic symptoms of LDDs include leg pain, numbness and other discomforts, intermittent claudication, and so on caused by lumbar disc herniation and lumbar spinal canal stenosis (lateral recess stenosis, intervertebral foramen stenosis, central spinal canal stenosis). Surgical treatment is needed for LDDs if the effects of conservative treatment are poor and the quality of life is seriously affected. Conventional open surgery has extensive soft tissue dissection, large trauma, much bleeding, a long postoperative recovery time, and a high incidence of complications (3–7). Moreover, the patients with LDDs are generally older, their tolerance of open surgery is poor, and the surgical risk is extremely high (8–12). How to reduce the surgical trauma of LDDs has become very important.

Minimally invasive spine surgery for treating LDDs is gradually being accepted, and spinal endoscopic surgery is one of the most minimally invasive techniques (13–15). In 2017, we first introduced our PTES (percutaneous transforaminal endoscopic surgery) technique (16) under local anesthesia with reduced steps, simple orientation, and easy puncture, which can significantly decrease the fluoroscopy projection time and shorten the operation time (17). It can effectively treat almost all kinds of LDDs. Additionally, different compressed lumbar nerve roots can lead to pain in the special place of the buttock and leg (14,18,19). Therefore, the involved nerve roots could be determined by the clinical symptoms, which might predict the culprit segment of LDDs (14,19,20). However, if the pain position of the buttock and leg is inconsistent with the levels and degrees of lumbar degeneration such as disc herniation, lateral recess stenosis, or intervertebral foramen stenosis on MRI and CT, should surgical treatment be performed according to radiologic images or nerve root symptoms? In this study, PTES under local anesthesia was performed to treat the culprit segment of LDDs predicted by radiologic images or clinical symptoms, and the efficacy, security, and feasibility were evaluated.

## Materials and methods

### Patients

**Figure 1:**
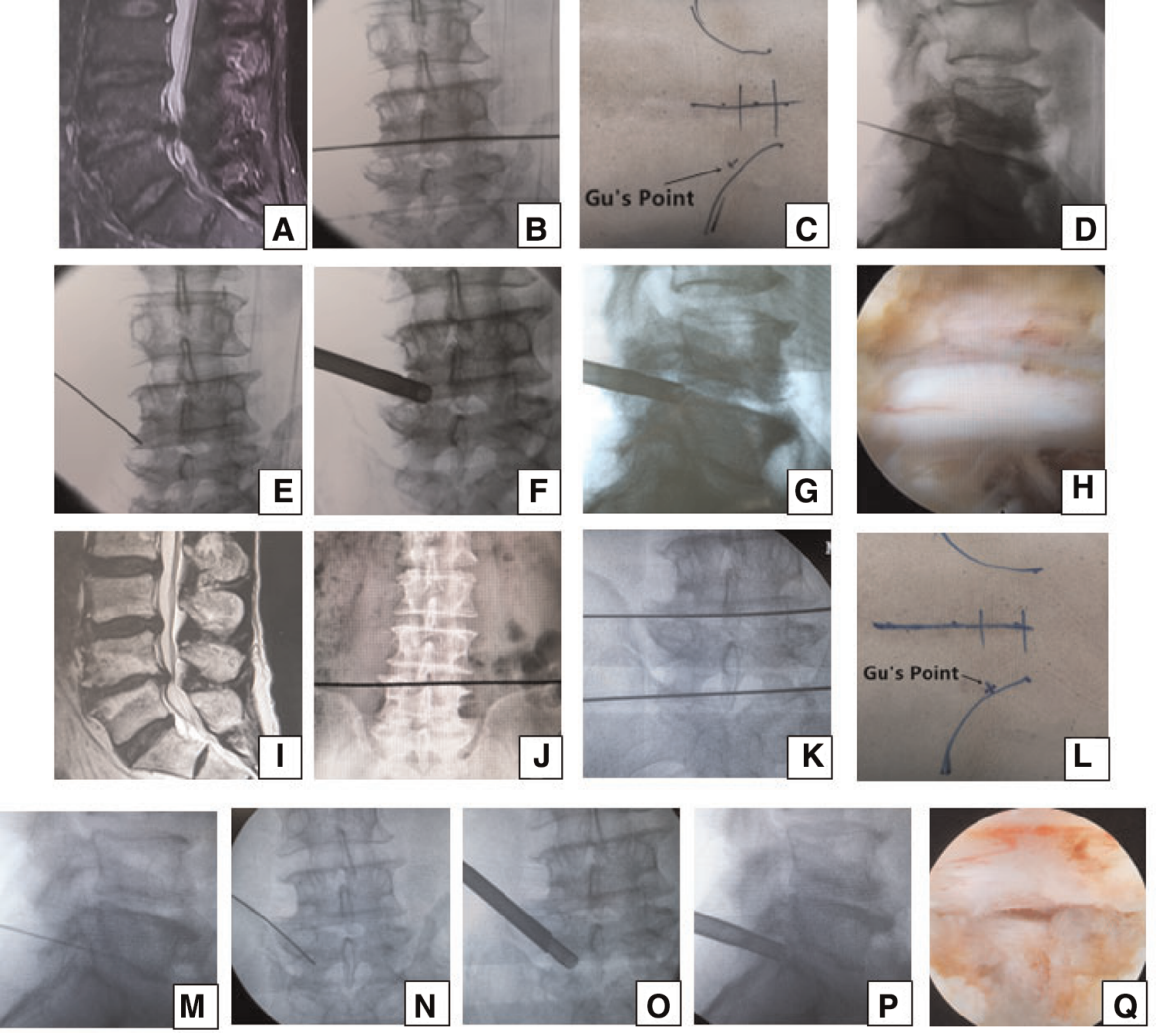
Male patient aged 76 years in group A had pain in the left part of the buttock and the posterior part of the thigh, calf and plantar preoperatively, which suggests that the S1 nerve root is involved and the culprit segment is generally L5/S1, but there were L4/5 massive disc herniation and lateral recess stenosis on (**A**) MRI. PTES was performed for L4/5, and the culprit segment was predicted according to radiologic images. A transverse line bisecting the disc was drawn along the metal rod, which was placed transversely across the center of the target disc on (**B**) posteroanterior C-arm view. (**C**) Photograph showing the surface marking of the anatomic disc center identified by the intersection of the transverse line and longitudinal midline and the entrance point of the puncture (“Gu's Point”) located at the corner of the flat back turning to the lateral side. After a successful puncture, the C-arm view was taken to ensure that the tip of the puncture needle was in the intracanal area close to the posterior wall of the disc on (**D**) lateral x-ray and near the lateral border of the pedicle on (E) posteroanterior x-ray. During press-down enlargement of the foramen, when resistance disappears, the tip of the reamer should exceed the medial border of the pedicle on (**F**) posteroanterior C-arm view and reach close to the posterior wall of the target disc on (**G**) lateral C-arm view. Under (**H**) endoscopic view, the nerve root was exposed after the ligamentum flavum and herniated disc were removed. During the operation, the patient gave a misleading response of involved leg relaxation and we did not undertake PTES for L5/S1. He had no relief from symptoms after surgery, and L4/5 degeneration improved on (**I**) MRI. The patient underwent another PTES for L5/S1 3 months after surgery. On (**J**) posteroanterior x-ray, the lower plate of the L4 vertebral body was not higher than the line between the highest points of the bilateral iliac crest, which is a high iliac crest. (**K**) Posteroanterior C-arm view was used to confirm the operation segment. (**L**) Photograph showing “Gu's Point” locating at the corner of the flat back turning to the lateral side. After a successful puncture, the tip of the puncture needle should be in the intracanal area close to the posterior wall of the disc on (**M**) lateral x-ray and near the lateral border of the pedicle on (**N**) posteroanterior x-ray. After the enlargement of the foramen, the tip of the reamer should exceed the medial border of the pedicle on (**O**) posteroanterior C-arm view and reach close to the posterior wall of the target disc on (**P**) lateral C-arm view. Under (**Q**) endoscopic view, the compressed nerve root was freed, and the patient had an obvious sense of relaxation in the left leg. He achieved a satisfying result after surgery.

**Figure 2:**
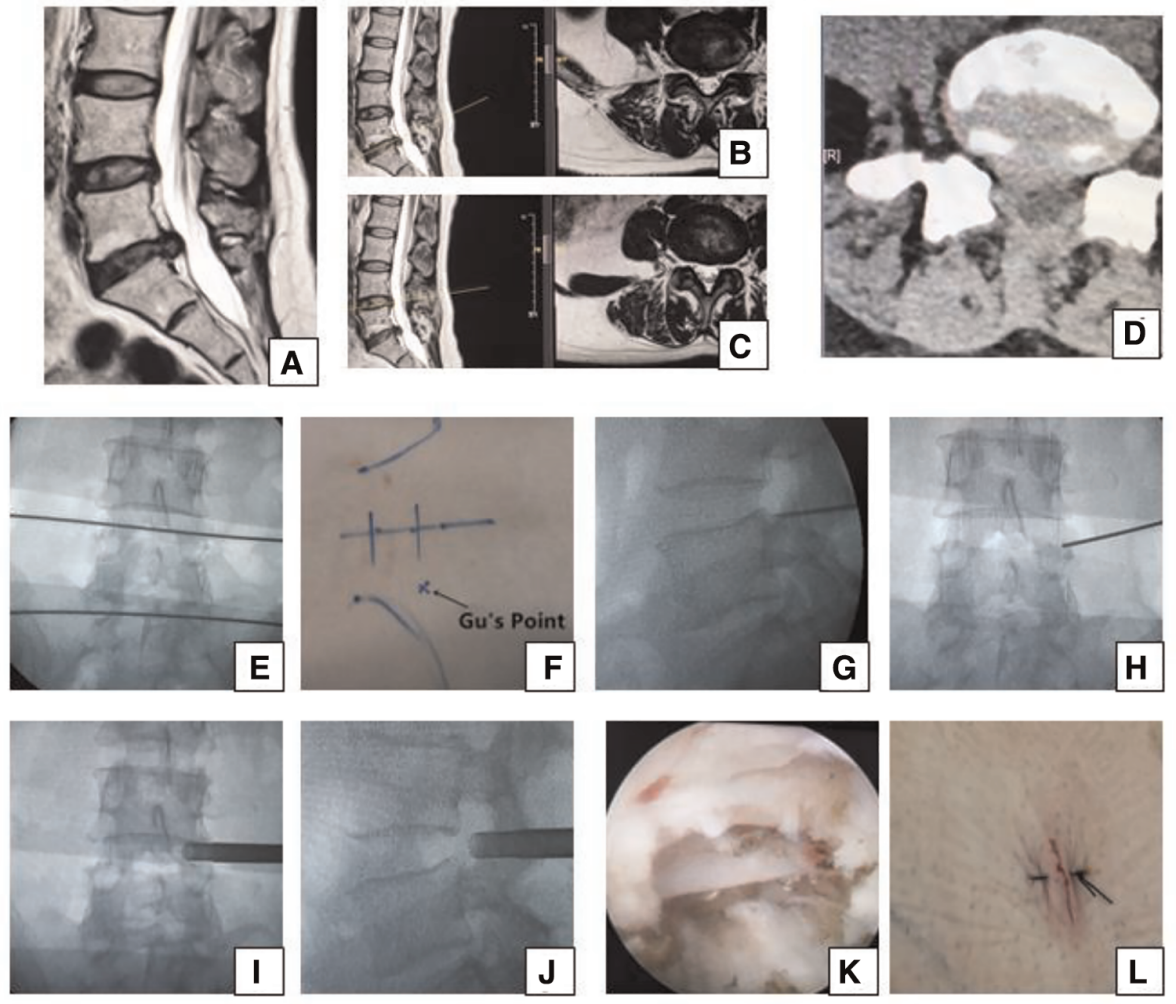
Female 50-year-old patient in group B had right leg pain from the posterolateral part of the buttock and thigh to the lateral part of the calf and the dorsal part of the foot, which indicates that the L5 nerve root is involved and the culprit segment is L4/5 (traversing nerve root) or L5/S1 (exiting nerve root). (**A**) Sagittal MRI, (**B,C**) axial MRI, and (**D**) axial CT showing L5/S1 huge disc herniation, which does not involve the right intervertebral foramen. We planned to perform PTES for L4/5. A transverse line bisecting the disc was drawn along the metal rod that was placed transversely across the center of the target disc on (**E**) posteroanterior C-arm view. (**F**) Photograph showing the surface marking of the anatomic disc center identified by the intersection of the transverse line and longitudinal midline, and Gu's Point located at the corner of the flat back turning to the lateral side. After a successful puncture, the tip of the puncture needle should be in the intracanal area close to the posterior wall of the disc on (**G**) lateral x-ray and near the lateral border of the pedicle on (**H**) posteroanterior x-ray. During press-down enlargement of the foramen, when resistance disappears, the tip of the reamer should exceed the medial border of the pedicle on (**I**) posteroanterior C-arm view and reach close to the posterior wall of the target disc on (**J**) lateral C-arm view. Under (**K**) endoscopic view, the nerve root was freed after the hypertrophic ligamentum flavum and herniated disc were removed. The patient had obvious relaxation in the right leg, and (**L**) the incision was closed. She achieved a satisfying result after surgery.

**Figure 3:**
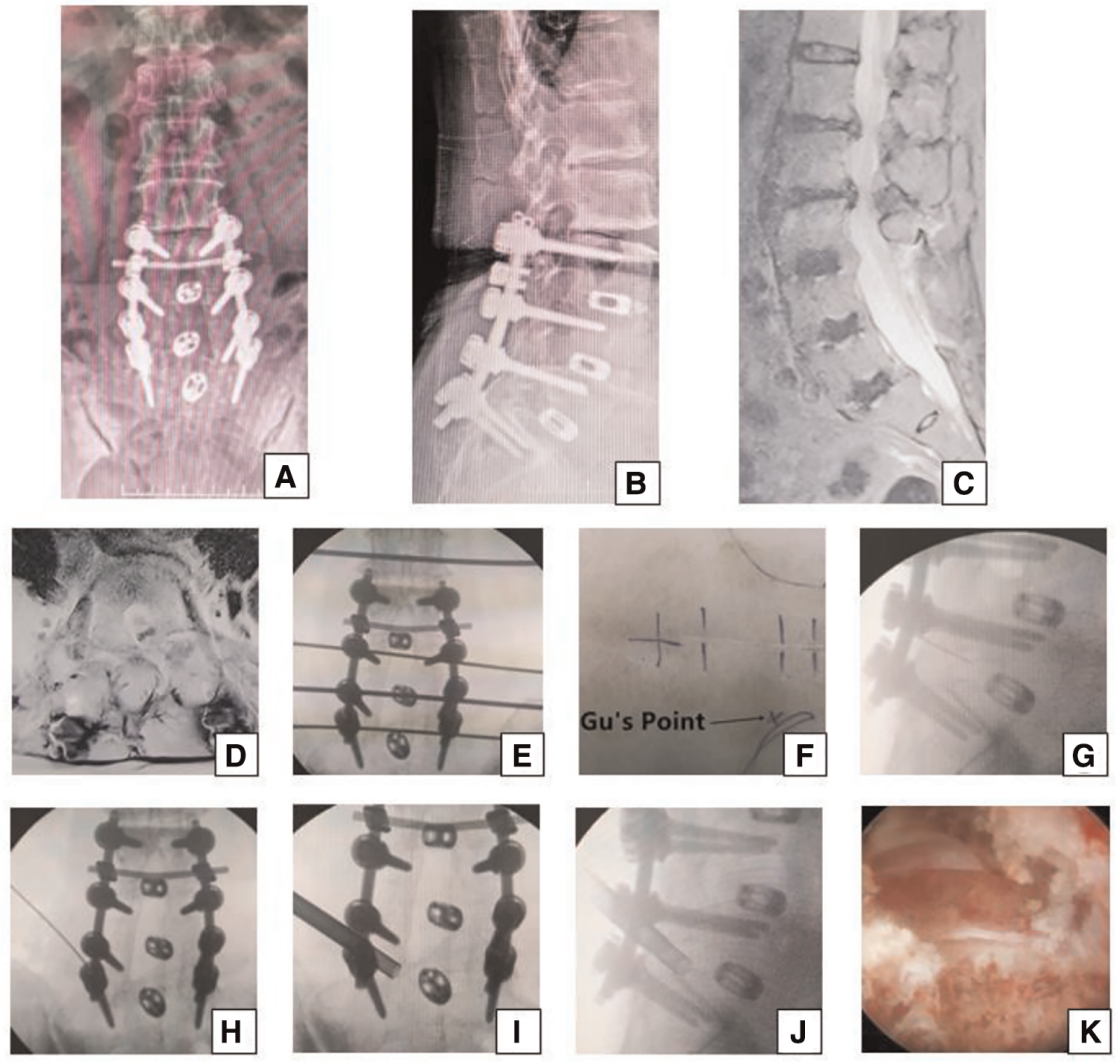
Female 74-year-old patient in group B underwent an open surgery of posterior decompress and fusion 13 years ago, which was shown on the (**A,B**) x-ray. She had pain in the left part of buttock and the posterior part of thigh, calf and plantar 12 years later, which suggests that the S1 nerve root is involved and the culprit segment is generally L5/S1. However, on (**C**) sagittal MRI and (**D**) L5/S1 axial MRI, the neurologic compression at L1/2, L2/3 with disc herniation, and lateral recess stenosis was more severe than that at L3/4 and L5/S1. The PTES procedure was undertaken for L5/S1. The transverse line bisecting the disc was drawn along the metal rod that was placed transversely across the center of the target disc on (**E**) posteroanterior C-arm view. (**F**) Photograph showing the surface marking of the anatomic disc center identified by the intersection of the transverse line and longitudinal midline, which was the aiming reference point of the puncture, and Gu's Point located at the corner of the flat back turning to the lateral side. After a successful puncture, the tip of the puncture needle should be in the intracanal area close to the posterior wall of the disc on (**G**) lateral x-ray and near the lateral border of the pedicle on (**H**) posteroanterior x-ray. After press-down enlargement of the foramen, the tip of the reamer should exceed the medial border of the pedicle on (**I**) posteroanterior C-arm view and reach close to the posterior wall of the target disc on (**J**) lateral C-arm view. Under (**K**) endoscopic view, the compressed nerve root was freed after the hyperplastic scars and osteophytes were removed. A satisfying result was achieved after surgery.

Eighty-seven cases of LDDs with nerve root symptoms, which were not consistent with lumbar degeneration levels and degrees on radiologic images (Figures 1A, 2A–D, 3A–D), were treated with PTES under local anesthesia in a day surgery ward from January 2015 to December 2019. They were followed up for more than 2 years. Forty-two patients, whose culprit segment was predicted by radiologic images, were included in group A. The other 45 patients, whose culprit segment was predicted by clinical symptoms, were included in group B. The patients enrolling depended on the surgeon's experience and the patient's selection. The detailed data are shown in Table 1. This retrospective cohort study was approved by the Ethics Committee of Zhongshan Hospital Fudan University.

**Table 1:** Demographic data of LDD patients whose culprit segments were predicted by radiologic images (A) or clinical symptoms (B).

	A (*n* = 42)	B (*n* = 45)	*p*-Value
Age	67.05 ± 11.96	67.24 ± 13.47	0.258^a^
Gender			0.948^b^
M	22	24	
F	20	21	
BMI	22.35 ± 2.70	23.29 ± 2.81	0.372^a^
Radiologic involved segment			0.478^b^
L1/2	0	1	
L2/3	10	8	
L3/4	28	32	
L4/5	41	41	
L5/S1	35	34	
			0.578^b^
1-Level	1	0	
2-Leve	21	25	
3-Level	11	15	
4-Level	9	5	
Accompanied degeneration			0.741^b^
Calcification	4	3	
Scoliosis	3	3	
High iliac crest (L5/S1)	2	3	
Lumbar fusion surgery history	0	1	
Surgical segment			0.0^b^
L1/2	0	0	
L2/3	0	0	
L3/4	14	5	
L4/5	41	25	
L5/S1	33	16	
			0.0^b^
1-Level	2	44	
2-Level	37	1	
3-Level	3	0	
Follow-up time (months)	49.79 ± 17.77	49.33 ± 16.47	0.863^a^

^a^ Exhibited as “mean ± standard deviation” and tested by Student's *t*-test.

^b^ Pearson's chi-squared test.

Inclusion criteria are as follows: (1) The nerve root symptoms are unilateral leg pain, bilateral asymmetric leg pain, or bilateral symmetric leg pain when at rest; (2) image data such as MRI and CT show lumbar degeneration from L1 to S1 including lumbar disc herniation, intervertebral foramen stenosis, or lateral recess stenosis (Figures 1A, 2A–D, and 3C,D), which are not consistent with the nerve root symptoms; (3) regular conservative treatment of at least 3 months has failed; (4) the systemic status is good, basic medical diseases such as heart disease, hypertension, or diabetes are under control, and the mental state is normal with independent understanding, thinking ability, and normal compliance; and (5) the patient can be followed up for at least 2 years.

Exclusion criteria are as follows: (1) intermittent claudication with no symptoms of legs when at rest and symmetric pain, numbness, discomfort, or tiredness of both legs after walking 50–100 m, unable to walk, relieved after rest, which is diagnosed as lumbar central spinal canal stenosis; (2) imaging examination showing lumbar spondylolisthesis and lumbarization of S1; (3) lumbar spine inflammation, tumors, and other lesions; (4) mental illness, coagulation dysfunction, and infection in the surgical area.

### Pre- and postoperative imaging

All patients were evaluated before the procedure by CT and MRI imaging to determine the involved segment or whether there was calcification. Posteroanterior and lateral radiographs were obtained to detect lumbar instability, scoliosis, lumbarization of S1, or high iliac crest when the lower plate of the L4 vertebral body was not higher than the line between the highest points of the bilateral iliac crest. After the treatment, MRI images were obtained to assess neurologic decompression or exclude dural cyst, myelomeningocele, dural tears or spinal fluid leaks, and reherniation.

### Surgical procedure

For group A, the culprit segment was predicted by radiologic images of MRI and CT.

For group B, the culprit segment was predicted according to the position of patient's leg pain. The central buttock, posterior thigh, posterior calf, lateral malleolus, or plantar: S1 nerve root is involved, and the culprit segment is generally L5/S1. The lateral buttock, posterolateral thigh, lateral calf, or dorsal foot: L5 nerve root is involved, and the culprit segment is generally L4/5 (traversing nerve root) or L5/S1 (exiting nerve root). The lateral buttock, anterolateral thigh, knee, medial calf, or medial malleolus: L4 nerve root is involved, and the culprit segment is generally L3/4 (traversing nerve root) or L4/5 (exiting nerve root). The distal one-third of the anterior thigh or the medial part of the condyle: L3 nerve root is involved, and the culprit segment is generally L2/3 (traversing nerve root) or L3/4 (exiting nerve root). The middle one-third of the anterior aspect of the thigh: L2 nerve root is involved, and the culprit segment is generally L1/2 (traversing nerve root) or L2/3 (exiting nerve root). The proximal one-third of the anterior aspect of the thigh: L1 nerve root is involved, and the culprit segment is generally T12/L1 (traversing nerve root) or L1/2 (exiting nerve root) (18–20). The first target is the segment involving traversing nerve root because the proportion of far lateral lumbar disc herniation or intervertebral foramen stenosis involving exiting nerve root in LDDs is relatively lower.

**Figure 4:**
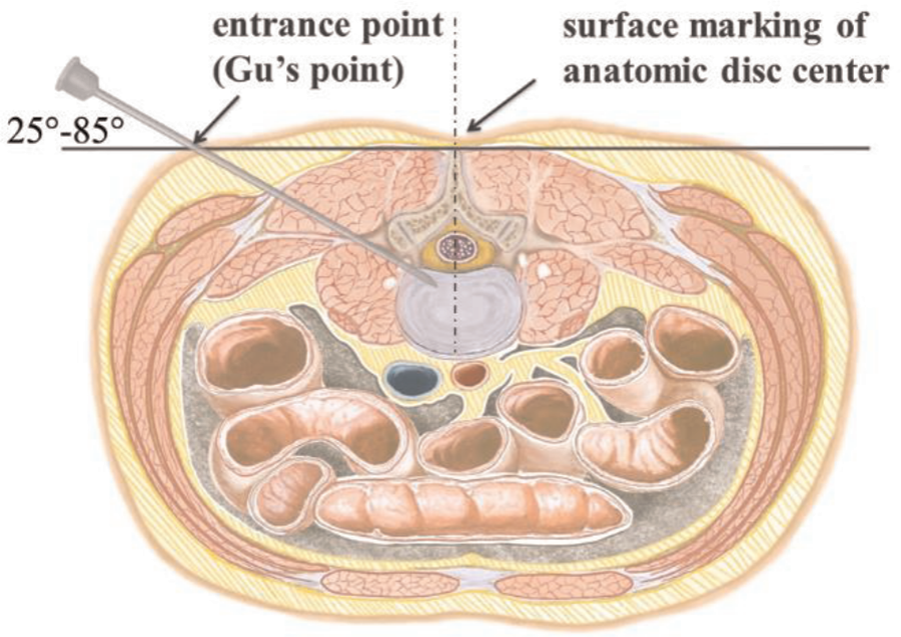
Perpendicular line through the surface projection of the anatomical center of intervertebral space is the target of the puncture. The entrance point of the puncture is located at the corner of the flat back turning to the lateral side, which is named “Gu's Point.” The puncture needle is inserted at a 25°–85° angle to the horizontal plane anteromedially.

**Figure 5:**
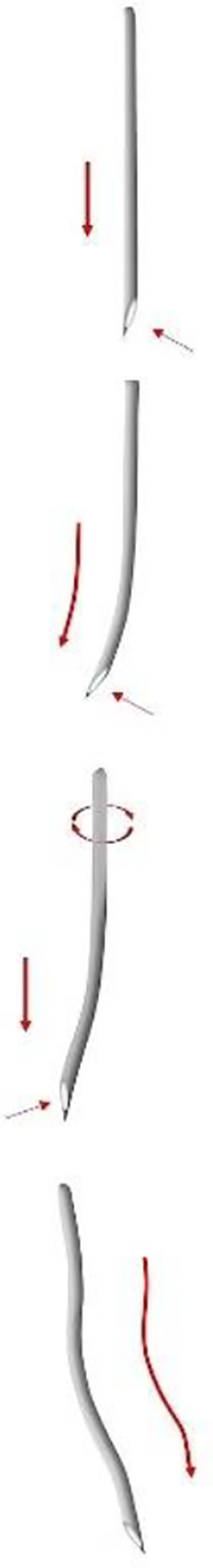
Minor adjustment of the puncture technique. Due to the principle that the needle moves forward in the opposite direction to the needle tip bevel, changing the direction of the needle tip bevel can adjust the puncture trajectory or even bypass the obstacle and reach the target segment.

**Figure 6:**
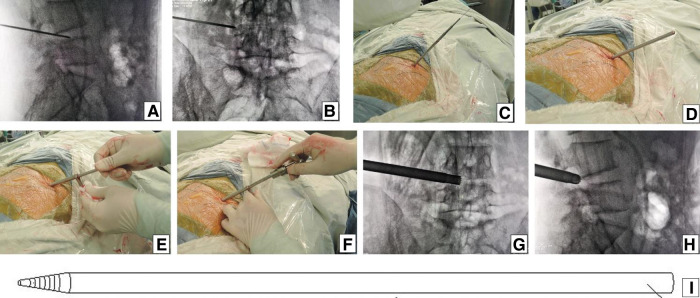
Guiding rod technique. After the puncture needle was inserted at 55° to the horizontal plane until the resistance disappeared, the C-arm view was taken to ensure that the tip of the puncture needle was in the posterior one-third of intervertebral space on (**A**) lateral x-ray and near the lateral border of the pedicle on (**B**) posteroanterior x-ray. (**C**) Over the guiding wire, stepwise-dilating cannulas were introduced to the anulus fibrosus beside the foramen. (**D**) Thick guiding rod of 6.3 mm diameter was inserted over the guiding wire and then adjusted into the foramen by moving the tip dorsally and cephalad after removing the guiding wire. (**E**) An 8.8-mm protective cannula was pushed over the rod to the facet joint area, docked at the facet, and pressed down. (**F**) A 7.5-mm reamer was introduced to remove the ventral bone of the articular process for enlarging the intervertebral foramen. When resistance disappears, the tip of reamer should exceed the medial border of the pedicle on (**G**) posteroanterior C-arm view and reach close to the posterior wall of the target disc on (**H**) lateral C-arm view. (**I**) Picture showing the stiff thick guiding rod of 6.3 mm diameter, which is easier to adjust the puncture trajectory than the soft puncture needle.

**Figure 7:**
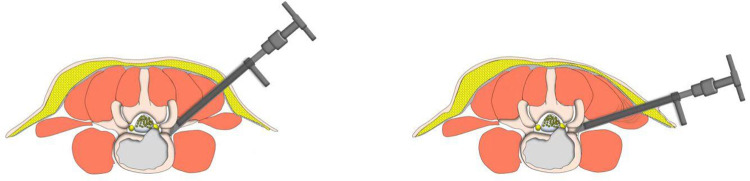
Cannula docked at the facet was pressed down further to remove more ventral parts of the upper articular process with a 7.5 mm reamer for further enlargement of the foramen and makes it easy to place the working cannula into the spinal canal between the dural sac and disc, which is press-down enlargement of the foramen.

PTES was performed under local anesthesia with 1% lidocaine supplemented with conscious sedation for the culprit segment. The patient was placed in a prone position with hyperkyphotic bolsters placed under the abdomen on a radiolucent table, especially in the cases of L5/S1 with a high iliac crest. The location of the culprit segment was determined by posteroanterior C-arm fluoroscopy (Figures 1B, 2E, 3E). The puncture point was located at the corner of the flat back turning to the lateral side according to “Gu's Point" (16, 17) (Figures 1C, 2F, 3F). The vertical line of the back surface was aimed through the intersection of the location line and midline and the puncture needle was inserted at 25°–85° to the horizontal plane (Figure 4). After a successful puncture, lateral C-arm fluoroscopy was performed to confirm that the tip of the puncture needle should reach the posterior one-third or near the posterior wall of the culprit intervertebral disc (Figures 1D, 2G, and 3G) and the posteroanterior film should be near the outer edge of the pedicle (Figures 1E, 2H, and 3F). If the puncture is not good, minor adjustment of needle position could be achieved based on the principle that the needle moves forward in the opposite direction to the needle tip bevel, which is a “minor adjustment of the puncture technique” (Figure 5). When the puncture trajectory was very difficult to adjust, a stiff guiding rod of 6.3 mm diameter could be used to adjust the direction more easily than the soft puncture needle. We termed it as the “guiding rod technique” (Figure 6). After the soft tissue was expanded step by step, an 8.8-mm protective cannula was inserted over the guiding rod, docked at the facet, and pressed down further to make the angle of the cannula to the horizontal plane smaller and a 7.5-mm reamer was introduced to remove the ventral bone of the articular process for enlarging the intervertebral foramen, which is “press-down enlargement of the foramen” (16, 17) (Figure 7). When resistance disappears, posteroanterior fluoroscopy shows that the tip of the reamer should exceed the inner edge of the pedicle and reach near the posterior wall of the target intervertebral disc on the lateral film (Figures 1F,G, 2I,J, and 3I,J). For lumbar central spinal canal stenosis, press-down enlargement of the foramen was repeated to enlarge the central spinal canal. The 7.5-mm working channel was placed along the guiding rod. Under the endoscopic vision, the hypertrophic ligamentum flavum and the protruding intervertebral disc tissue were removed to enlarge the lateral recess, and the ipsilateral traversing nerve root, exiting nerve root, or epidural sac were exposed and decompressed (Figures 1H, 2K, 3K). For the patient with bilateral asymmetric leg pain, we performed PTES from the side of more severe leg pain to achieve bilateral decompression. The “press-down enlargement of the foramen” technique made it easy to remove the herniated disc underneath the ipsilateral nerve root, the central dura, and even the contralateral nerve root. The operation of two adjacent segments could be completed through one surgical incision (Figure 2L).

During the operation under local anesthesia, communication with the patients was made to confirm the efficacy. When the patient was placed in a prone position, there were generally no symptoms of leg pain and numbness, but the involved legs had an obvious sense of relaxation after neurologic decompression was achieved. We used the visual analogue scale (VAS) to evaluate the relaxation sensation of the involved leg. Preoperative status of no relaxation is 10, and complete relaxation is 0: 0–3, obvious relaxation, good outcome, the treated segment is the culprit one, and the operation can be finished; 4–6, moderate relaxation, partially effective, the treated segment is the culprit one, and other culprit segments need treatment; 7–10, mild relaxation or no relaxation, no efficacy, the treated segment is not the culprit one, and the culprit segment needs treatment.

The blood loss is calculated as follows. The blood absorption capacity in one piece of dry gauze is about 30 ml. The blood loss is calculated by the proportion of red area in the total area of gauze. The blood loss under endoscopy is calculated as the difference in the outflow and inflow volumes of the irrigation solution. The sum of the above two is total blood loss during PTES.

### Postoperative care

After the operation, the patients rested in bed until the next day and then walked with a flexible back brace for 2 weeks. The functional exercise began on the third day after the operation, and the patients went to work 1 week after the operation.

### Clinical follow-up

VAS was used to score the leg pain before the operation and immediately, 1 month, 2 months, 3 months, 6 months, 1 year, and 2 years after the operation. ODI scores before PTES and 2 years after PTES were recorded. At a 2-year follow-up, the MacNab grade was used to evaluate the results: excellent, good, moderate, or poor.

### Statistical analysis

SPSS 25 software (SPSS Inc., Chicago, IL, USA) was used to perform statistical analysis. Normal distributed continuous variables such as age, BMI, incision length, follow-up, and ODI score were presented as mean ± standard deviation (SD); discrete, rating, and continuous variables, including operative duration, fluoroscopy frequency, blood loss, hospital stay, and VAS, which are not normally distributed, were presented as median (maximum−minimum); categorical variables such as gender, lumbar level, and rates of calcified herniation, scoliosis, lumbarization of S1, high iliac crest, and rates of excellent and good outcomes were expressed as frequency or percentage. Student’s *t*-test was used for the intergroup analysis of normally distributed continuous variables. The Mann–Whitney *U* test was used for the intergroup analysis of discrete variables, rating variables, and continuous variables, which are not normally distributed. Pearson's chi-squared test was used for the intergroup analysis of unordered categorical variables, and Fisher's exact test was used for ordered categorical variables. Intragroup comparison of leg pain VAS at different time points was conducted using a linear mixed-effects model for group A and the Kruskal–Wallis test followed by the Dunn procedure with Bonferroni correction for group B. Preoperative and postoperative ODI scores were compared using Student’s *t*-test. *p* < 0.05 was considered a significant difference.

## Results

Table 1 summarizes the baseline data of two groups. There is no statistical significance in age, gender, BMI, preoperative involved segments on MRI and CT, and follow-up time between the two groups. There are four patients with calcified herniation, three with scoliosis, and two with a high iliac crest (L5/S1) in group A, and three patients with calcified herniation, three with scoliosis, three with a high iliac crest (L5/S1), and one with lumbar fusion surgery history in group B. The operation-related data are shown in Table 2. In group A, 2 patients underwent PTES for one segment, 37 patients underwent PTES for two segments, and 3 patients underwent PTES for three segments. One of the one-segment PTES patients had no relief from symptoms and underwent another PTES for other segments 3 months after surgery (Figures 1I–Q). In group B, 44 of 45 patients were treated using PTES for one segment and 1 patient were treated for two segments. Group B showed significantly less operative duration from the body position to incision closure (108.5/52–164 min vs. 55/46–101 min, *p* < 0.001), less blood loss (14/4–35 ml vs. 6/3–12 ml, *p* < 0.001), and low fluoroscopy frequency (13/5–21 times vs. 6/5–11 times, *p* < 0.001) than group A. All procedures were performed through the unilateral approach and only one incision was needed for two segments adjacent to each other.

**Table 2:** Operation-related data of both two groups.

	Group A	Group B	*p*-Value
Operative duration (min)	108.5 (52–164)	55 (46–101)	<0.001^a^
Frequency of fluoroscopy (times)	13 (5–21)	6 (5–11)	<0.001^a^
Blood loss (ml)	14 (4–35)	6 (3–12)	<0.001^a^
Incision length (mm)	9.5 ± 1.9	9.6 ± 1.7	<0.001^b^
One incision (case)	38	45	
Two incisions (case)	4	0	
Hospital stay (days)	3 (2–4)	3 (2–3)	0.687^a^

^a^ Exhibited as “median (min−max)” and tested by the Mann–Whitney *U* test.

^b^ Exhibited as “mean ± standard deviation” and tested by Student's *t*-test.

The leg pain VAS scores of groups A and B decreased from 8 (7–10) before surgery to 1 (0–7) and 1 (0–2) (*p* < 0.001) immediately after surgery, further decreasing to 0 (0–1) and 0 (0–1) (*p* < 0.001) 2 years after surgery, respectively. The leg pain VAS score of group B was significantly lower than that of group A immediately, 1 week, 1 month, 2 months, and 3 months after surgery (*p* < 0.001), but there was no statistical difference between the two groups 6 months, 1 year, and 2 years after surgery. Five patients (11.9%, 5/42) in group A and one patient (2.2%, 1/45) in group B had a rebound effect of leg pain (16, 17, 21). VAS scores of these patients increased 1 week after surgery, and the pain got relieved within 2 months. The preoperative ODI scores of groups A and B significantly decreased from 72.1 ± 9.7% and 66.7 ± 8.7% to 15.5 ± 5.0% and 13.2 ± 4.6% (*p* < 0.001) at 2-year follow-up, respectively (Table 3). According to the MacNab classification, the excellent and good rate was 97.6% (41/42) for group A and 100% (45/45) for group B 2 years after surgery (Table 4). There was no statistical difference in ODI scores and the excellent and good rates between the two groups. No complications such as wound infection, permanent nerve injury, abdominal organ injury, large vessel rupture, and recurrence were observed.

**Table 3:** Leg pain VAS and ODI scores of both groups.

	Group	Before surgery	After surgery							
			Immediately	1 week	1 month	2 months	3 months	6 months	12 months	24 months
VAS score	A	8 (7–10)	1 (0–7)^b^	1 (0–8)^b^	1 (0–8)^b^	1 (0–8)^b^	1 (0–8)^b^	0 (0–1)^bcde^	0 (0–1)^bcde^	0 (0–1)^bcde^
	B	8 (7–10)	1 (0–2)^ab^	1 (0–7)^ab^	0 (0–7)^ab^	0 (0–2)^abcd^	0 (0–1)^abcde^	0 (0–1)^bcde^	0 (0–1)^bcde^	0 (0–1)^bcde^
ODI score	A	72.1 ± 9.7								15.5 ± 5.0^b^
	B	66.7 ± 8.7								13.2 ± 4.6^b^

^a^ *p* < 0.001, compared with group A.

^b^ *p* < 0.001, compared with preoperatively.

^c^ *p*< 0.001, compared with immediately after surgery.

^d^ *p* < 0.05 compared with 1 week after surgery.

^e^ *p* < 0.05 compared with 1 month after surgery.

**Table 4:** MacNab classification data at 24 months after surgery.

	Group A	Group B
Excellent	32	39
Good	9	6
Fair	0	0
Poor	1	0
Excellent or good rate	97.6%	100%
*p*-Value^a^	0.597	

^a^ Excellent or good rate is tested by Fisher's exact test.

## Discussion

In practice, we found that most patients with LDDs have unilateral leg pain or asymmetric pain in both legs and few have symmetric pain in both legs when at rest, all of which are symptoms of nerve root compression and should be diagnosed as lumbar disc herniation, lateral recess stenosis, or intervertebral foramen stenosis, although radiological imaging showed lumbar central spinal canal stenosis in some cases. When we used PTES to treat LDDs with nerve root symptoms, press-down enlargement of the foramen was performed to remove the ventral bone of the facet joint and made the working channel enter into the spinal canal between the traversing nerve root and disc (16, 17). In addition, the hypertrophic ligamentum flavum and the protruding nucleus pulposus were removed to enlarge the lateral recess and decompress the nerve root. The ipsilateral and contralateral traversing nerve roots could be exposed, and the bilateral nerve roots could be decompressed from one side through a small incision. During the PTES procedure under local anesthesia, we used VAS to evaluate the relaxation sensation of the involved leg after neurologic decompression. A VAS score of 0–3 means obvious relaxation and good efficacy, the treated segment is the culprit one, and the operation can be finished. A VAS score of 4–6 indicates moderate relaxation, the treatment is partially effective, and the treated segment is the culprit one, but other culprit segments need treatment. A VAS score of 7–10 shows mild or no relaxation and no efficacy, the treated segment is not the culprit one, and the culprit segment needs treatment until the VAS score decreases to 0–3. This method can guarantee surgical efficacy. The results of this study showed that the leg pain VAS score and ODI score significantly decreased after the operation (*p* < 0.001) in both groups, and the excellent and good rate was 97.6% in group A and 100% in group B at the 2-year follow-up. Interestingly, a 74-year-old female patient in group B underwent an open surgery of L3–S1 posterior decompression and fusion (Figures 3A,B) 13 years ago, and 12 years later, she felt left leg pain again without lumbar instability and cage migration. After the PTES procedure for L5/S1 (Figures 3E–K), a satisfying result was achieved, which indicates that the nerve root might have been compressed by the hyperplastic scars and osteophytes, although the spinal canal and lateral recess were opened, and the facet joint and disc were removed for the insertion of the cage.

It is very important to predict the culprit segment in the surgical treatment of LDDs, especially multilevel (≥2 levels) lumbar degenerations on MRI or CT, and the culprit segment leading to symptoms is often only one of them (22–27). In general, we predict the culprit segment according to lumbar degeneration and neurologic compression on MRI and CT. Additionally, the culprit segment can be predicted according to the position of leg pain (14, 18–20). If radiologic images of MRI and CT show the segments of lumbar disc herniation, intervertebral foramen stenosis, and lateral recess stenosis, which is consistent with the nerve root symptoms, there is no controversy for the culprit segment.How to predict the culprit segment when radiologic images are not in accordance with neurologic symptoms? Some scholars suggest that the nerve root block test is helpful (28, 29). The nerve root block imparts effect through local anesthesia medicine, so it is not sure that the injection site must be the level of neurologic compression. Sometimes, it is difficult for the nerve root block test to determine the culprit segments when both traversing and exiting nerve roots are blocked at the same segment or the blocked nerve root is compressed at another segment. In group B of this study, 44 of 45 patients after PTES for one segment achieved satisfying results, which confirms that the prediction of the culprit segment according to the position of leg pain is relatively accurate. Only one patient was treated using PTES for two segments because two nerve roots were involved. In group A, PTES was performed for the segment showing the most severe degeneration on MRI and CT, 40 of 42 patients had no significant relief from symptoms or obvious relaxation of involved legs during the operation, another segment was treated in 37 patients, and other two segments were treated in 3 patients until the efficacy was achieved. These indicate that radiologic images are not reliable in predicting culprit segments. Only two patients of group A underwent PTES for one segment, and they had pain in the posterior part of the buttock, thigh, calf and plantar preoperatively, which suggests that the S1 nerve root is involved and the culprit segment is generally L5/S1, but there were L4/5 massive disc herniation and lateral recess stenosis on MRI and CT. Of them, the male patient aged 76 years had no relief from symptoms after PTES for L4/5 and underwent another PTES for L5/S1 3 months after surgery and achieved a good result. This maybe because he gave a misleading response of involved leg relaxation due to dysaudia, influencing the communication, and we did not undertake PTES for L5/S1 during the first operation. The other male 40-year-old patient had leg pain relief after PTES for L4/5, possibly due to the huge disc herniation compressing the next traversing nerve root of S1, which is rare in our experience. The results of this study showed that group B of culprit segment prediction according to neurologic symptoms had significantly less operative duration, less blood loss, and low fluoroscopy frequency than group A of culprit segment prediction according to radiologic images. In addition, the rebound effect of leg pain (16, 17, 21) occurred in five patients (11.9%, 5/42) of group A and one patient (2.2%, 1/45) of group B, which indicates that the stimulation on the nerve elements might induce the neurologic symptoms, especially at the segment of severe degeneration on MRI and CT without clinical symptoms before surgery.

**Figure 8:**
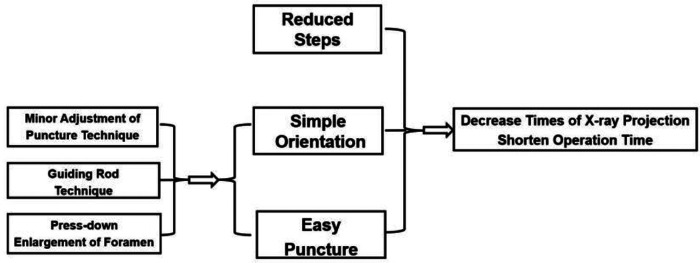
Simple orientation and easy puncture are achievable because of three crucial techniques in PTES including “minor adjustment of puncture technique,” “guiding rod technique,” and “press-down enlargement of foramen,” which could significantly decrease the exposure of x-ray and the operation time.

In our PTES technique, orientation was simple, we only needed to perform posteroanterior fluoroscopy to determine the horizontal line of the culprit segment (Figures 1B, 2E, and 3E). The vertical line through the intersection point of the horizontal line and midline of the back (anatomical center of the intervertebral disc) was the target of the puncture (Figure 4). The entrance point of the puncture was located at the corner of the flat back turning to the lateral side, which does not need to measure the distance lateral to the midline. We named it “Gu's Point” (16, 17) (Figures 1C, 2F, and 3F), which is closer to the midline than that in other posterolateral endoscopic surgeries such as TESS, and there are four advantages. (1) It avoids the exiting nerve root. If the entrance point locates laterally, the exiting nerve root may be more possibly met. (2) It avoids blockage by the high iliac crest for the L5/S1 level. The height of the iliac crest at “Gu's Point” is relatively shorter, reducing the difficulty of puncture and subsequent operation (Figures 1J,L). (3) It shortens the manipulation path in obese patients. The more lateral from the midline the entrance point, the longer the path for the surgical target. Especially more subcutaneous adipose tissue of obese patients makes the puncture point more distal from the surgical target, which needs a very long working channel for transforaminal endoscopic surgery. (4) It avoids abdominal viscera and main blood vessels. Puncture from “Gu's Point” is much safer, and the tip of the needle could be blocked by the bony structure of the spine. In PTES, the puncture is easy, and it is acceptable that the tip of the needle is in the posterior one-third of the intervertebral space on the lateral C-arm view once the needle reaches the target. Simple orientation and easy puncture are achievable because of three crucial techniques in PTES, including the “minor adjustment of the puncture technique,” “guiding rod technique,” and “press-down enlargement of the foramen” technique. Due to the principle that the needle moves forward in the opposite direction to the needle tip bevel, changing the direction of the needle tip bevel can adjust the puncture trajectory or even bypass the obstacle and reach the target segment. This is the “minor adjustment of the puncture technique” (Figure 5). It is easier for the stiff guiding rod to adjust the puncture trajectory than the soft puncture needle, which is the “guiding rod technique” (Figure 6). Sometimes the facet joint is very hard and the reamer skids during the foramen enlargement, which can be solved by rotating the reamer along the guiding rod inserted into the intervertebral foramen. Although the puncture needle enters the intervertebral disc, we press down the protective cannula anchored against the facet joint and make its angle to the horizontal plane smaller for enlarging the foramen, which can let the reamer remove the ventral side of the facet joint and makes it easy to place the working cannula into the spinal canal between the dural sac and disc. It is called “press-down enlargement of the foramen” (16, 17) (Figure 7). In PTES, a 7.5-mm reamer is used to enlarge the foramen in one step instead of step by step. This reduced steps, simple orientation, and easy puncture can significantly decrease the frequency of fluoroscopy projection and shorten the operation time (Figure 8).

There are also some limitations of this study. It is a single-center retrospective study with a relatively small number of patients. There is no comparison of PTES with other techniques, such as MIS-TLIF. Therefore, we will perform a multicenter prospective controlled study.

## Conclusion

It is much more accurate to predict the culprit segment according to clinical symptoms than radiologic images in PTES under local anesthesia for surgical treatment of LDDs, which can decrease the operative duration, blood loss, fluoroscopy frequency, and postoperative rebound effect of leg pain. The entrance point of PTES (Gu's Point) is located at the corner of the flat back turning to the lateral side, and in PTES, there are three crucial techniques of “minor adjustment of the puncture technique,” “guiding rod technique,” and “press-down enlargement of the foramen.” PTES is not only a minimally invasive surgical technique but also includes a preoperative and intraoperative assessment system for guaranteeing operative efficacy.

## Data Availability

The raw data supporting the conclusions of this article will be made available by the authors without undue reservation.
